# Base-modified Donor Analogues Reveal Novel Dynamic Features of a Glycosyltransferase*

**DOI:** 10.1074/jbc.M113.465963

**Published:** 2013-07-08

**Authors:** René Jørgensen, Thomas Pesnot, Ho Jun Lee, Monica M. Palcic, Gerd K. Wagner

**Affiliations:** From the ‡ Department of Microbiology and Infection Control, Statens Serum Institut, DK-2300 Copenhagen S, Denmark,; the § Carlsberg Laboratory, Gamle Carlsberg Vej 10, DK-1799 Copenhagen V, Denmark,; the ¶ University of East Anglia, School of Pharmacy, Norwich NR47TJ, United Kingdom,; the ‖ Department of Chemistry, University of Alberta, Edmonton, Alberta T6G2G2, Canada, and; the ** King's College London, School of Biomedical Sciences, Institute of Pharmaceutical Science and Department of Chemistry, Franklin-Wilkins Building, 150 Stamford Street, London SE1 9NH, United Kingdom

**Keywords:** Crystallography, Enzyme Inhibitors, Glycosyltransferases, Kinetics, Molecular Dynamics

## Abstract

Glycosyltransferases (GTs) are enzymes that are involved, as Nature's “glycosylation reagents,” in many fundamental biological processes including cell adhesion and blood group biosynthesis. Although of similar importance to that of other large enzyme families such as protein kinases and proteases, the undisputed potential of GTs for chemical biology and drug discovery has remained largely unrealized to date. This is due, at least in part, to a relative lack of GT inhibitors and tool compounds for structural, mechanistic, and cellular studies. In this study, we have used a novel class of GT donor analogues to obtain new structural and enzymological information for a representative blood group GT. These analogues interfere with the folding of an internal loop and the C terminus, which are essential for catalysis. Our experiments have led to the discovery of an entirely new active site folding mode for this enzyme family, which can be targeted in inhibitor development, similar to the DFG motif in protein kinases. Taken together, our results provide new insights into substrate binding, dynamics, and utilization in this important enzyme family, which can very likely be harnessed for the rational development of new GT inhibitors and probes.

## Teaser

**Background:** Modified UDP-Gal donor substrates with 5-formylthienyl and 5-phenyl substituents on the uracil base exhibit differential inhibition patterns for glycosyltransferases.

**Results:** Structural studies reveal a new enzyme loop folding mode for the 5-formylthienyl analogue.

**Conclusion:** Differential inhibition is attributed to alternate enzyme conformational changes and interactions with the respective inhibitors.

**Significance:** The conformational plasticity of glycosyltransferases can be exploited in designing novel inhibitors.

## Introduction

**FIGURE 1.**
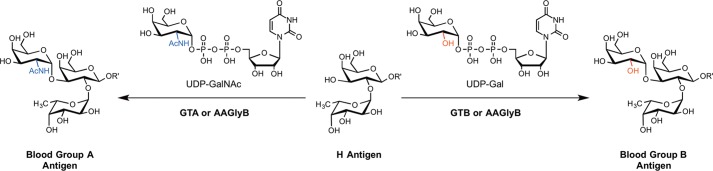
**Reactions catalyzed by blood group glycosyltransferases GTA, GTB, and AAGlyB.**

GTs^3^ are a large class of enzymes that catalyze glycosidic bond formation by the transfer of a mono- or oligosaccharide from a glycosyl donor, usually a sugar-nucleotide, to an appropriate acceptor (typically another sugar, lipid, protein or small molecule) (1, 2). These enzymes are responsible for producing simple and complex oligo- and polysaccharides as well as glycoconjugates, such as glycoproteins and glycolipids. Due to the ubiquitous presence of saccharides and glycoconjugates in all domains of life, GTs are critically involved in many fundamental biological processes. One important example is the biosynthesis of human ABO blood group antigens, which is the most important blood group system in transfusion and transplantation. Structurally, the blood group A and B epitopes differ in only a single sugar (A: *N*-acetyl d-galactosamine; B: d-galactose). The respective sugar is installed at the common H antigen core by one of two GTs, GTA and GTB (Fig. 1). GTA catalyzes the transfer of GalNAc from UDP-GalNAc to the H antigen acceptor (HAA) (α-l-Fuc-(1→2)-β-d-Gal-*O*-R, where R is glycolipid or glycoprotein) to form the A antigen, whereas GTB catalyzes the transfer of Gal from UDP-Gal to the HAA to form the B antigen. GTA and GTB are highly homologous, differing in only 4 amino acids (Arg/Gly-176, Gly/Ser-235, Leu/Met-266, Gly/Ala-268), yet crucially, display different donor substrate specificities (3). Naturally occurring mutants of GTA and GTB include rare *cis*-AB enzymes that produce both blood group A and B structures. Among these is the enzyme AAGlyB, a dual-specificity GT, which can bind and utilize either UDP-Gal or UDP-GalNAc with equal efficiency (Fig. 1) (3, 4). A nomenclature based on the four critical residues has been developed to describe GTA and GTB chimera, where GTA can be referred to as AAAA and GTB as BBBB. Each letter corresponds to one critical residue in increasing order such that the AAGlyB chimer would correspond to GTA-L266G/G268A. The blood group enzymes are among the best structurally characterized GTs and can thus serve as excellent models for mechanistic studies in this enzyme family and for the development of specific GT inhibitors.

Previous kinetic and structural studies on the blood group GTs suggest that the UDP-sugar donor substrate binds first to an “open” form of the enzyme (5, 6). Reorganization of an internal flexible loop (residues 173–188) concomitant with donor binding generates a “semiclosed” state and creates an acceptor binding site. Upon binding of the acceptor substrate, the enzyme adopts a “closed” conformation in which the final nine C-terminal amino acid residues become ordered by forming hydrogen bonds to both UDP and acceptor (5). Such extensive conformational rearrangements during the enzyme catalytic cycle are characteristic for GTs in general and have been observed for many other enzymes in this family (7).

**FIGURE 2.**
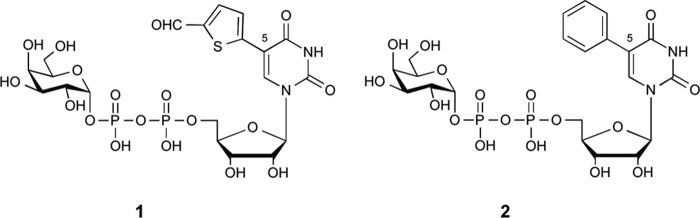
**Structures of base-modified UDP-Gal donor analogues used in this study.**

The development of new chemical tools for the investigation of GT mechanism and structure as well as GT-dependent processes in cells is of considerable interest for biological chemistry and chemical biology (8–11). These efforts are hampered by uncertainty about GT reaction mechanisms and the numerous conformational changes that occur upon substrate binding and during the catalytic cycle (2, 7). Using AAGlyB as a model system, we have recently developed a new class of GT donor analogues derived from UDP-Gal, the natural donor substrate of galactosyltransferases (GalTs) (8). The new derivatives are characterized by an additional substituent in position 5 of the uracil base, as exemplified by prototype analogue **1** (Fig. 2). Intriguingly, this modification turned **1** into a poor donor substrate, but a potent inhibitor for AAGlyB, with very low turnover rates (8). Crystallographic and enzymological studies with **1** and AAGlyB provided some insights into the structural basis for this intriguing behavior. An initial structure of AAGlyB in complex with **1** raised the possibility that the 5-formylthienyl substituent in **1** may be wedged between residue Trp-181 of the flexible active site loop and Arg-352 in the C terminus (8). In this position, the 5-formylthienyl substituent would block the stacking of the two residues Trp-181 and Arg-352. This stacking interaction would normally stabilize the closed conformation of the enzyme, which contributes to the formation of the acceptor binding site and is required for full catalytic activity. Interference with this crucial conformational transition therefore appeared as a possible explanation for the inhibitory activity of **1** toward AAGlyB and several other GalTs (8, 12), as the movement of a flexible active site loop is a very common motif during catalysis in the GT family (7).

Importantly, however, in our previously reported structure of the AAGlyB-**1** complex, an 8-residue stretch in the flexible loop, including Trp-181, as well as the last 8 residues of the C terminus, including Arg-352, were not resolved. This structure could therefore not provide an exact explanation at the molecular level for this new allosteric mode of GT inhibition. It also failed to answer several other significant questions: Does the 5-formylthien-2-yl substituent in **1** interact directly with flexible loop residues, and if so, how? Why are alternative 5-substituents, such as 5-phenyl (compound **2**, Fig. 2), less effective at inhibition? (12) Why does the enzyme retain low residual catalytic activity? We anticipated that answers to these questions would not only allow the rational optimization of this new class of GalT inhibitors, but also provide insight into fundamental aspects of GT mechanisms.

To address these questions, we have carried out additional structural and enzymological studies with two base-modified UDP-Gal derivatives with two different 5-substituents (Fig. 1, compounds **1** and **2)**. Herein, we report four new structures of AAGlyB in complex with, respectively, compound **1** and the HAA substrate α-l-Fuc*p*-(1→2)-β-d-Gal*p*, compound **2**, **2** and HAA, and, finally, UDP and HAA. In conjunction with detailed enzymological data, these structures provide an explanation for the different binding affinities and turnover rates of the two UDP-Gal derivatives **1** and **2**, which have different 5-substituents. Intriguingly, the ternary complex structure AAGlyB-**1**-HAA reveals an alternative closed conformation of the C terminus, which has not previously been observed, and which can account for the better binding of **1** compared with **2** and the residual rate of product formation highlighting the conformational plasticity of GTs.

## EXPERIMENTAL PROCEDURES

### 

#### 

##### General

UDP-Gal and UDP-GalNAc were obtained from Pharma-Waldhof GmbH (Duesseldorf) and Sigma, respectively, and used as received (purity ≥97%). All other biochemicals were obtained commercially and used as received unless stated otherwise.

##### Synthesis

The UDP-Gal derivatives **1** (8) and **2** (12) were synthesized as reported previously.

##### Cloning, Purification, and Crystallization

The mutant enzyme was cloned and expressed in *Escherichia coli* using standard mutagenesis and expression techniques (13, 14). Briefly, the AAGlyB mutant (GTA-L266G/G268A) was constructed by PCR using a GTA-G268A mutant clone (AAAB) as a template (15). The forward primer MIN2 (5′-ATA TGA ATT CAT GGT TTC CCT GCC GCG TAT GGT TTA CCC GCA GCC GAA-3′) introduced an EcoRI site at the 5′ end, and the reverse primer PCR3B (5′-ATA ATT AAG CTT CTA TCA CGG GTT ACG AAC AGC CTG GTG GTT TTT-3′) introduced a HindIII site at the 3′ end of the gene. Two fragments were amplified with *Pfx* DNA polymerase (Invitrogen) by using the forward primer MIN2 together with HJL06 (5′-GAA AGC ACC ACC GTA GTA GAA GTC ACC TTC G-3′) and the reverse primer PCR3B with HJL07 (5′-C TAC TAC GGT GGT GCT TTC TTC GGT GGT TCC-3′). HJL06 and HJL07 were designed so that the two fragments overlapped each other and have a single codon substitution (CTG to GGT) at codon 266. The two overlapping fragments were isolated, annealed by 3′ extension by using PCR and amplified by using the outside primers MIN2 and PCR3B. The amplified genes were digested by restriction enzymes (EcoRI, HindIII) and ligated into the previously digested pCWΔlac vector (16). The ligation reaction was incubated at room temperature overnight and transformed into *E. coli* BL21-gold using CaCl_2_-competent cells. A single transformant was inoculated into LB broth containing ampicillin and incubated overnight at 37 °C. Plasmids were purified with a mini plasmid preparation column. The entire sequence was confirmed by sequencing using a DYEnamic ET terminator cycle sequencing kit. AAGlyB was purified by ion-exchange (SP-Sepharose) and affinity chromatography (UDP-hexanolamine-Sepharose eluted with 5 mm free UDP) as described (17) and yielded ∼15 mg of pure protein/liter of cell culture. At the end of purification, excess UDP was removed from the eluted protein solution by dialysis in 50 mm MOPS, pH 7, 0.1 m NaCl, 1 mm DTT, 5 mm MnCl_2_ before concentrating the protein to 15 mg/ml using a Vivaspin 20 3,000 MWCO (Sartorius). The mutant enzyme was crystallized as described previously (8). Crystals of the individual AAGlyB-donor analogue complexes were flash frozen in liquid N_2_ after a cryosolution-containing reservoir solution, 20% glycerol and 25 mm concentration of the respective donor analogue **1** and **2** was added to a drop with crystals and then soaked for 30 min. The AAGlyB-donor analogue-HAA crystals were flash frozen in liquid N_2_ as for AAGlyB-donor analogue crystals but in a cryosolution also containing 25 mm acceptor. The AAGlyB-UDP-HAA structure was solved as an attempt to soak a donor analogue and HAA into a crystal where UDP removal after the final step in the purification had been insufficient.

##### Enzyme Kinetics

The *K_m_*, *k*_cat_, and *K_i_* values for UDP-Gal, UDP-GalNAc, **1**, and α-Fuc*p*-(1→2)-β-Gal*p-O*-(CH_2_)_7_-CH_3_ acceptor with AAGlyB have been reported previously (8). The *K_i_* value for **2** with AAGlyB was determined by a standard radiochemical assay, using a Sep-Pak reverse-phase cartridge to isolate radiolabeled reaction products as described previously (5). Because turnover of compound **2** is negligible, it can be evaluated as a competitive inhibitor in radiochemical assays. The *K_i_* value was obtained by linear regression analysis of a Dixon plot using 100 μm HAA, 2 μm UDP-Gal, and 0, 2, 4, or 8 μm **2**. The *K_m_* and *k*_cat_ values for **2** and α-Fuc*p*-(1→2)-β- Gal*p-O*-(CH_2_)_8_CONH(CH_2_)_2_NH-CO-tetramethylrhodamine acceptor (18) were determined using capillary electrophoresis (CE) to separate and measure labeled reaction products from labeled acceptor. For donor kinetics with **2**, **2** (3.9–250 μm), AAGlyB protein (4 μg/ml), labeled acceptor (50 μm), MnCl_2_ (20 mm), BSA (1 mg/ml), and MOPS buffer (50 mm, pH 7.0) were incubated for 60 min at 37 °C (total volume 12 μl, all concentrations are final concentrations). For acceptor kinetics with **2**, the following concentrations were used: **2** (33.3 μm) and labeled acceptor (500–7.8 μm). The reactions were stopped by adding 348 μl of borate buffer (5 mm, pH 9.3) containing SDS (15 mm). CE was performed using an automated PrinCE 2-lift, model 560 CE system (Prince Technologies). Separations were carried out in an uncoated fused silica capillary of internal diameter 75 μm with an effective length between 40 and 75 cm (plus 30 cm extra from the detection window to outlet), thermostatically controlled at 25 °C. The CE running buffer was 50 mm borate buffer, pH 9.3, containing 150 mm SDS. The capillary was initially conditioned by rinsing at 2000 mbar for 20 min with 1 m NaOH, 10 min with water, and 10 min with background electrolyte. Between runs the capillary was washed at 2000 mbar for 3 min with 1 m NaOH, 3 min with water, and 3 min with background electrolyte. Detection was carried out using a 532-nm laser ZETALIF Evolution detector (LIF VI-01, Picometrics). Samples were injected hydrodynamically for 6 s at 50 mbar and electrophoresed across a potential difference of 25 kV. All experiments were carried out at a normal polarity, *i.e.* inlet anodic. *K_m_* and *V*_max_ values were determined by fitting data points to a Michaelis-Menten curve using GraphPad PRISM 4 (GraphPad Software). Control experiments carried out in the absence of enzyme (to account for potential chemical hydrolysis) showed no significant degree of degradation on the time scale of the enzymatic experiments. To verify that kinetic values obtained by CE measurements are comparable with those obtained by the radiochemical assay described above we determined the *k*_cat_ values for UDP-Gal and UDP-GalNAc under saturating conditions in parallel assays using both methods.

##### Data Collection, Reduction, and Structure Solution

For the AAGlyB-**1**-HAA and AAGlyB-**2**-HAA complex structures x-ray diffraction data to 1.9 Å and 1.7 Å resolution, respectively, were collected at beamline I911-2 at the MAXII-lab in Lund Sweden (λ = 1.038 Å, 100 K). Data on the AAGlyB-**2** complex were collected to 1.75 Å resolution at MAXII-lab beamline I911-5 (λ = 0.908 Å, 100 K). Finally, data on the AAGlyB-UDP-HAA complex were collected to 1.8 Å resolution at beamline ID-23-1 at the ESRF, Grenoble, France (λ = 0.979 Å, 100 K). All data sets were integrated and scaled by XDS (19). After integration and scaling the structures were solved by molecular replacement using the CCP4i module Phaser (20) and the previously solved structure of wild-type GTB (PDB ID code 2RIT) as a search model. The AAGlyB-**2** structure has one molecule in the asymmetric unit and space group C222_1_. The three other structures have two molecules in the asymmetric unit and space group P2_1_2_1_2. Initial rigid body and restrained refinement was carried out using REFMAC5 (21) before iteratively rebuilding the structure using Coot (22) and finally refining in PHENIX (23) including Translation/Libration/Screw vibrational motion (24). Ramachandran plots according to PROCHECK (25) show that within all four structures all residues are within the allowed region. The details of data collection and refinement for the enzyme complexes are provided in Table 1. All structural figures were prepared in PyMOL (DeLano Scientific LLG). A difference map of the AAGlyB-**2** complex shows a long and significant blob of electron density between two symmetry-related molecules (data not shown). The density most likely originates from PEG-3350 being present in the crystallization solution, however, attempts to model and refine a PEG molecule into the density failed.

**TABLE 1:** **Statistics for data collection and refinement**

**Crystal parameters**				
Complex	**2**	**2**-HAA	**1**-HAA	UDP-HAA
Beamline	MAXII I911-5	MAXII I911-2	MAXII I911-2	ESRF ID-23-1
Symmetry	C222_1_	P2_1_2_1_2		
Unit cell dimensions (Å)				
*a*	152.43	78.17	78.30	78.10
*b*	48.58	153.25	153.52	153.67
*c*	78.80	52.72	52.83	52.50

**Data collection**				
Resolution range (Å)	20.0–1.75 (1.72–1.68)	30–1.68 (1.95–1.90)	30.0–1.9	20.0–1.80 (1.90–1.80)
*R*_sym_*^b^* (%)	9.4 (65.0)	6.7 (68.8)	12.1 (65.2)	11.4 (65.6)
Completeness (%)	99.9 (99.9)	95.8 (90.3)	97.5 (95.5)	99.1 (99.4)
Average I/σ (I)	15.1 (3.3)	17.5 (2.2)	12.6 (2.7)	11.8 (2.5)
Redundancy	7.3 (7.4)	5.9 (4.8)	6.0 (5.2)	5.2 (5.3)

**Refinement statistics**				
Resolution range (Å)	19.7–1.75	29.05–1.68	29.11/1.90	19.94/1.80
Reflections				
Work set/Test set	30,505/945	67,183/2,799	47,821/1,987	57,223/1,771
No. of atoms				
Protein	2,308	4,741	4,836	4,930
Ligand	43	124	126	112
Waters	206	551	568	636
Glycerol				18
SO_4_	5	5	5	10
Overall *B*-factor (Å^2^)	21.9	21.9	18.1	17.1
R.m.s. deviation*^c^*				
Bond lengths (Å)	0.021	0.010	0.007	0.008
Bond angles (°)	1.884	1.300	1.049	1.208
*R*-factor*^d^* (%)	14.7	16.4	15.9	15.1
*R*_free_-factor*^e^* (%)	17.8	19.2	19.3	19.1
Ramachandran plot (%)				
Most favored	92.6	92.2	91.5	92.9
Additionally allowed	7.4	7.8	8.5	7.1

**Protein Data Bank ID**	3V0L	3V0M	3V0P	3V0Q

*^a^* Values in parentheses are for the highest resolution shell.

*^b^ R*_sym_ = Σ|(*I* − <*I*>)|*I*Σ(*I*), where *I* is the observed intensity.

*^c^* R.m.s., root mean square.

*^d^ r* = Σ‖*F_obs_*| − | *F_calc_*‖Σ|*F_obs_*|, where |*F_obs_*| and |*F_calc_*| are observed and calculated structure factor amplitude.

*^e^* The *R*_free_ value was calculated with a random 5% subset of all reflections excluded from refinement.

## RESULTS

### 

#### 

##### Enzyme Kinetics

Previously, we have determined the kinetic parameters for the glycosyl transfer reaction of AAGlyB with UDP-Gal derivative **1** as donor and the HAA acceptor by a standard radiochemical assay (Table 2) (8). These results showed that **1** is a good binder, but a poor substrate for AAGlyB. Whereas **1** has a *K_m_* similar to that of the natural donor substrates UDP-Gal and UDP-GalNAc, *k*_cat_ for the glycosyl transfer reaction was decreased ∼10–15-fold, and the *K_m_* for acceptor increased about 10-fold with **1** as a donor. In this study, we used a CE with tetramethylrhodamine-labeled HAA acceptor to determine the *K_m_* and *k*_cat_ values of the new donor analogue **2** with AAGlyB. This assay allowed us to follow the reaction by directly detecting the trisaccharide HAA reaction product. This type of assay gives values comparable with the radiochemical Sep-Pak reverse phase assay as shown by two identical assays set up in parallel and measured with the two different methods. The radiochemical assay gave results almost identical to the already reported *k*_cat_ values for UDP-Gal and UDP-GalNAc, and the *k*_cat_ values from CE are reported in Table 2. Interestingly, the enzymological profile of **2** is markedly different from that of **1** (Table 2). UDP-Gal derivative **2**, which differs from **1** in the nature of the 5-substituent, has a *K_m_* value that is approximately 10-fold higher than that of both the parent UDP-Gal and UDP-Gal derivative **1**. The *k*_cat_ of **2** is 1–2% of UDP-Gal and lower than that of **1**, by a factor of 4. These results suggest that the 5-phenyl substituent in **2** is less well tolerated at the donor binding site of AAGlyB than the 5-formylthienyl substituent in **1** and disfavors the binding of donor analogue **2** in a catalytically productive orientation. Finally, we also evaluated the potential inhibitory effect of **2** on AAGlyB in a radiochemical assay. Whereas both donor analogues were able to block glycosyl transfer under these conditions, **2** showed a 6-fold higher *K_i_* value than **1**, consistent with its higher *K_m_* value compared with **1**.

**TABLE 2:** **Enzymological characterization of UDP-Gal, UDP-GalNAc, and compounds 1 and 2 with AAGlyB**

Kinetics	UDP-Gal*^a^*	UDP-GalNAc*^a^*	1*^a^*	2
*K_m_* donor (μm)	0.7 ± 0.1*^b^*	1.7 ± 0.2*^b^*	<0.4*^c^*	6.5 ± 0.5*^d^*
	0.7 ± 0.06*^c^*			
*K_m_* acceptor (μm)	21 ± 2*^e^*	7.9 ± 0.6*^e^*	211 ± 24*^c^*	56 ± 3*^d^*
*k*_cat_ (s^−1^)	0.37 ± 0.06*^b^*	0.88 ± 0.07*^b^*	0.024 ± 0.001*^c^*	0.006 ± 0.0008*^d^*
	0.25 ± 0.01*^c^*	0.89 ± 0.01*^d^*		
	0.67 ± 0.06*^d^*			
*K_i_* (μm)	NA*^f^*	NA*^f^*	0.53*^b^*^,^*^g^*	3.0*^b^*^,^*^g^*
			0.52*^b^*^,^*^h^*	

*^a^* Kinetic values, except for the CE *k*_cat_ values, were previously reported in Ref. 8.

*^b^* Radiochemical assay, with 100 μm acceptor.

*^c^* HPLC assay.

*^d^* CE assay with tetramethylrhodamine-labeled HAA.

*^e^* Radiochemical assay, with 100 μm donor.

*^f^* NA, not applicable.

*^g^* Donor: UDP-Gal (2 μm).

*^h^* Donor: UDP-GalNAc (2 μm).

##### Crystallography

To investigate the structural basis for the different enzymological profiles of the closely related UDP-sugars **1** and **2**, structural studies were carried out with the analogues and AAGlyB, both in the presence and absence of acceptor. We have solved three new crystal structures of AAGlyB in complex with, respectively, **1** (donor analogue) and HAA (acceptor), **2**, and both **2** and HAA. For direct comparison, we also solved the structure of AAGlyB in complex with UDP and HAA. The details of data collection and refinement for the enzyme complexes are provided under “Experimental Procedures” and in Table 1. The maximum resolution of the diffraction data varied from 1.9 to 1.68 Å with final *R*_free_ values ranging from 17.8 to 19.3%. Three of the structures belong to space group P2_1_2_1_2 containing 2 molecules in the asymmetric unit whereas the fourth one, the AAGlyB-**2** complex, belongs to space group C222_1_ with only one molecule in the asymmetric unit. All four structures have similar unit cell parameters. The overall conformation of the four structures is highly isomorphous with the primary distinguishing characteristics restricted to the flexible internal loop from residue 173 to 188 and to the last 10 residues of the C terminus (residue 345–354). The core root means square deviation values between all four structures are between 0.102 and 0.673 Å as calculated by the SSM Superpose tool in Coot using the C_α_ atoms of all residues possible in each chain. The single chain in the AAGlyB-**2** structure shares the least similarity to the other structures.

##### AAGlyB Structure in Complex with UDP and HAA

**FIGURE 3.**
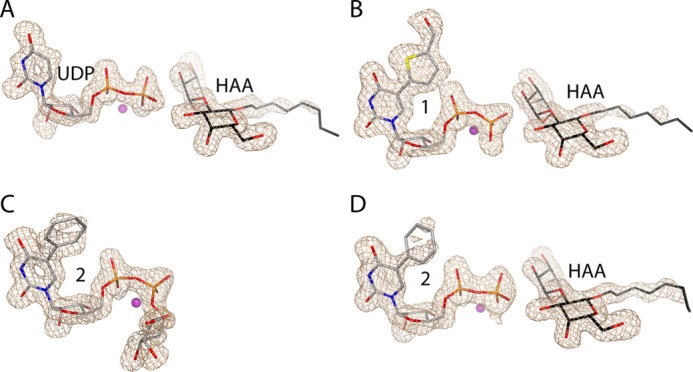
**Electron density maps of the UDP-Gal derivatives and HAA in the AAGlyB complex structures.** *A*, chain B of the AAGlyB-UDP-HAA structure. *B*, chain A of AAGlyB-**1**-HAA. *C*, chain A of AAGlyB-**2**. *D*, chain B of AAGlyB-**2**-HAA. All maps are *F_o_* − *F_c_* electron density simulated annealing omit maps (contoured at 3 σ) surrounding the UDP-Gal derivatives and HAA.

**FIGURE 4.**
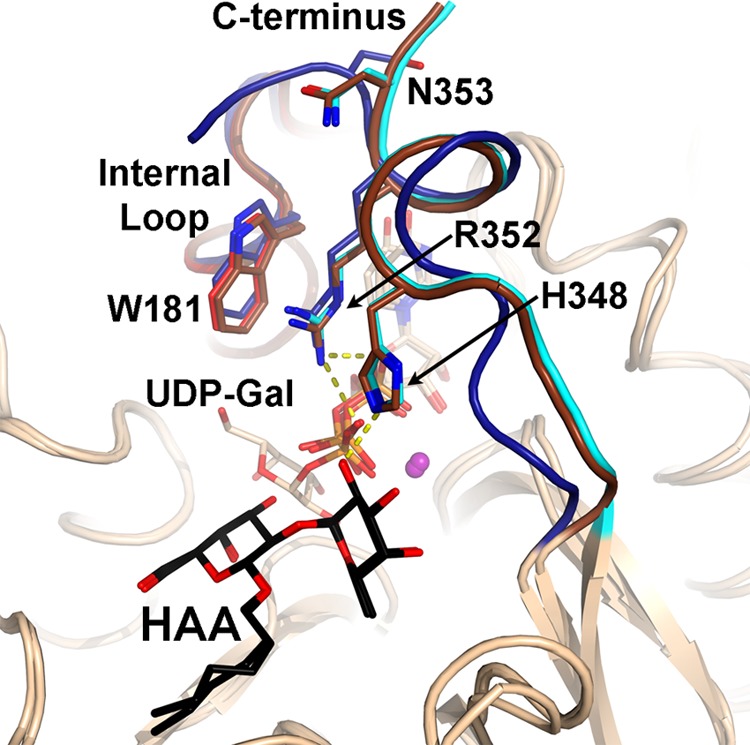
**Conformation of the C terminus in the fully closed state.** Overlay of the new AAGlyB-UDP-HAA complex structure (C terminus shown in *cyan*) with the previously solved structures of AABB-UDP-Gal-DA (PDB ID code 2RJ7) (C terminus shown in *brown*) and α-1,3-GalT-UDP (PDB ID code 1K4V) (C terminus shown in *blue*). Hydrogen bonds are shown in *yellow dashed lines*. The UDP-Gal and HAA are shown in *light brown* and *black sticks*, respectively.

The AAGlyB-UDP-HAA structure shows very well defined electron density for residues 58 to 354 of both chain A and B in the asymmetric unit including density for both HAA and UDP (Fig. 3*A*). Generally, GTs are known to have one or two flexible loops which undergo a significant conformational change from an open to a closed state upon substrate binding (7). It has previously been shown that for human blood group GTs the internal loop goes from an open to a semiclosed conformation upon donor binding, and when acceptor binds, the C terminus folds over the binding pocket to form the fully closed conformation (5, 7). In the new AAGlyB-UDP-HAA structure and the previously solved structure of the GTA-L266M/G268A (AABB) mutant in complex with UDP-Gal and the 3-deoxy-Fuc-3-amino-Gal-H antigen acceptor (DA) (5), the enzyme adopts this fully closed conformation where the active site loop and the C terminus fold over the active site (Fig. 4). As seen in both chain A and B of the AAGlyB-UDP-HAA structure the C terminus curls up into a short α-helix from residue 347 to 352 that makes contact with residues in the active site. Residue Arg-352 forms salt bridges with both phosphates of UDP-Gal and is stacking to Trp-181 in the internal loop as the only direct contact between the internal loop and the C terminus. Also, His-348 is forming a H-bond to the fucose moiety of the acceptor. A similar conformation of the C terminus is also seen in the crystal structure of the homologous α-1,3-GT in complex with UDP (Fig. 4) (26).

##### AAGlyB Structures in Complex with 1 and 1-HAA

**FIGURE 5.**
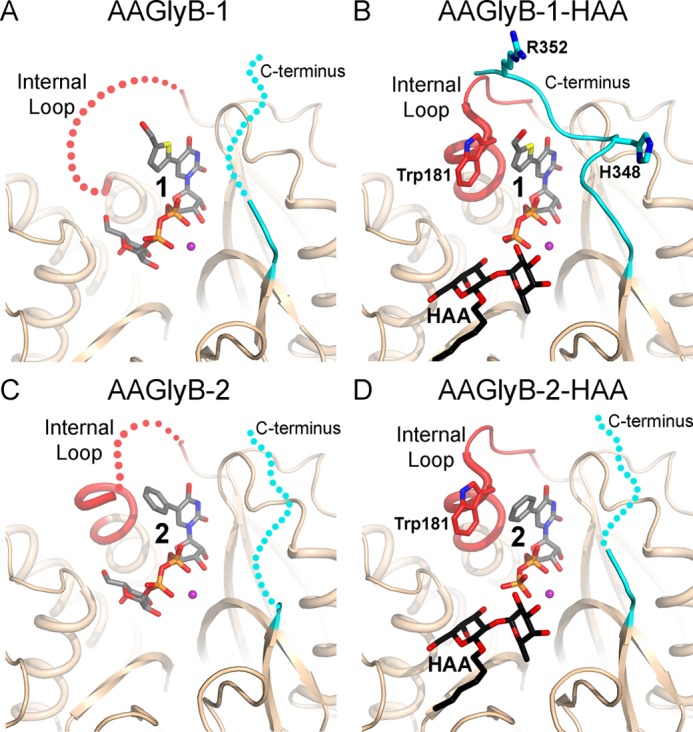
**Crystal structures of AAGlyB in complex with 1 and 2 with and without acceptor.** *A*, the AAGlyB-**1** complex (PDB ID code 1IOI) showing a disordered internal loop (*red*) and C terminus (*cyan*). Disordering is illustrated by *dotted lines*. Compound **1** including the Gal is shown in *gray carbon atoms. B*, AAGlyB-**1**-HAA complex showing an ordered internal loop in the closed conformation and the ordered C terminus. *Colors* are as in *A*. The acceptor is shown in *black carbon atoms. C*, AAGlyB-**2** complex. *D*, AAGlyB-**2**-HAA complex.

**FIGURE 6.**
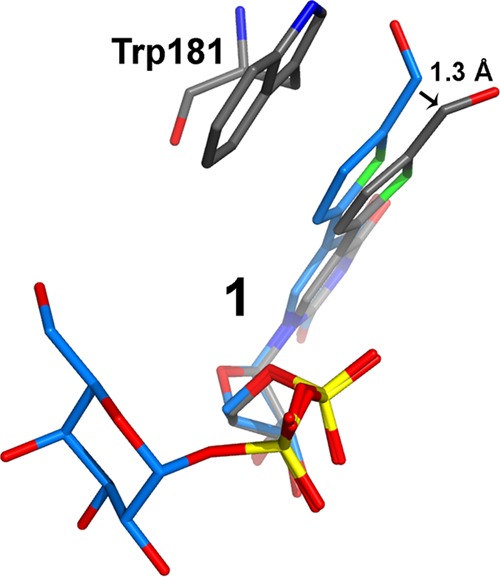
**Movement of the formylthienyl-substituent forced by Trp-181 during the internal loop closure. 1** from the AAGlyB-**1** structure is shown in *blue*. **1** and Trp-181 from the AAGlyB-**1**-HAA structure are shown in *gray*.

**FIGURE 7.**
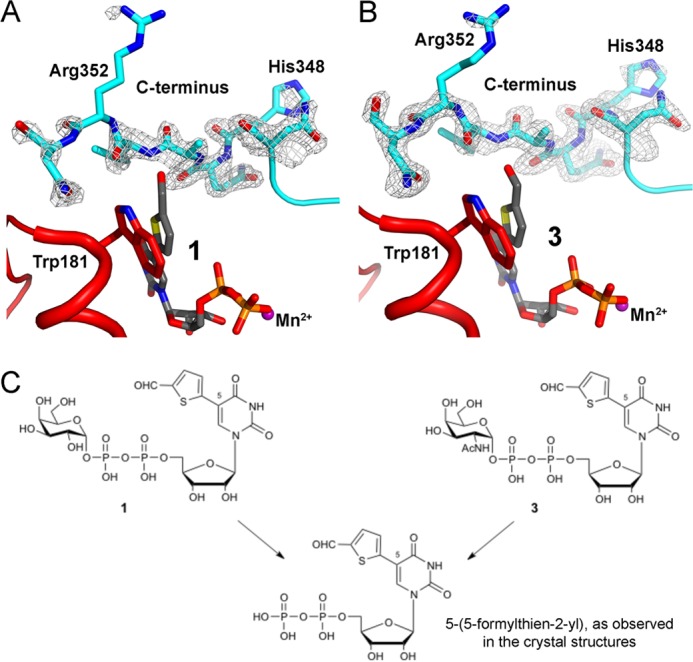
**Electron density map of the AAGlyB C terminus.** *A*, C terminus of the AAGlyB-**1**-HAA complex. A *F_o_* − *F_c_* electron density simulated annealing omit map surrounding residues 347–353 (contoured at 2.5 σ). *B*, C terminus of an identical structure with the same unit cell parameters and space group but soaked with a GalNAc congener of **1**, called **3**. Map type and contour level are the same as in *A*. Full details of the AAGlyB-**3**-HAA structure will be published elsewhere. *C*, chemical drawing showing the relationship among **1**, **3**, and the corresponding UDP derivative. Soaking with **3** leads to the same 5-formylthienyl UDP hydrolysis product. From the two structures it is clear that the C terminus adopts the same orientation in both structures, even if the electron density is somewhat weaker in the structure derived from **1**.

**FIGURE 8.**
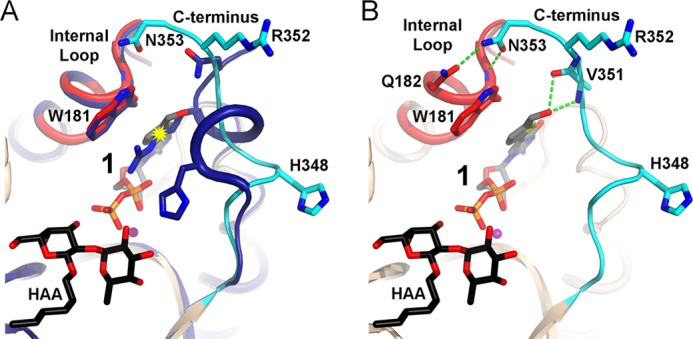
**Alternative conformation of the C terminus.** *A*, overlay of the AAGlyB-UDP-HAA (*dark blue*) and AAGlyB-**1**-HAA complexes showing the alternative conformation of the C terminus (*cyan*) in the structure with **1** (*gray carbon atoms*). Internal loop is shown in *red*. The potential clashing between Arg-352 and the substituent on **1** is marked by a *yellow starburst. B*, hydrogen bond formation (*green dashed lines*) of the C terminus to the substituent and the internal loop. *Colors* are as in *A*.

In the previously solved 1.45 Å structure of the AAGlyB-**1** complex, the internal active site loop from residue 177 to 186 and the C terminus from 346 to 354 were disordered (Fig. 5*A*) (8). Despite the high resolution, the structure provided no specific information about the conformation of the flexible loop in the presence of **1**, or any potential molecular interaction between loop residues and the 5-substituent of this donor analogue. The previous structure was obtained in the absence of acceptor, thus in the present study we soaked HAA acceptor into AAGlyB crystals together with **1** and Mn^2+^. The solved ternary structure shows well defined density for both HAA and **1** including the 5-substituent at the uracil base (Fig. 3*B*). However, except for a small and poorly defined electron density blob at the position of the galactose residue in chain B (data not shown) there is no density for the sugar, meaning that the galactose is either highly flexible or has been cleaved off from **1** and has not been transferred to the HAA acceptor. This is in contrast to the AAGlyB-**1** complex structure, which showed a fully defined **1** with a galactose in the catalytically competent “tucked under” conformation as previously described for the AABB-UDP-Gal-DA structure. Importantly, the entire active site loop in both chain A and B of the asymmetric unit as well as the C terminus of chain A are well resolved in the AAGlyB-**1**-HAA structure and in a conformation that has not previously been observed. The C terminus of chain B is visible only up until residue 346. The structure shows that the internal loop goes from a disordered state in the structure containing only **1** (Fig. 5*A*), to an ordered, closed conformation in the AAGlyB-**1**-HAA structure (Fig. 5*B*). However, in this new conformation, Trp-181 in the active site loop is not stacking to Arg-352 in the C terminus, as in the closed conformation with UDP and HAA (Fig. 4), but to the formythienyl substituent of **1**. Compared with AAGlyB-**1** this loop closure forces the formylthienyl substituent to be pushed over by about 1.3 Å to accommodate the bulky side chain of Trp-181 when the internal loop is folding over the active site (Fig. 6). Moreover, the C terminus of chain A has adopted an ordered state up until residue 353 (Figs. 5*B* and 7, *A–C*), which is folded over the donor binding site in a conformation alternative to that observed with the natural substrate. When comparing the AAGlyB-UDP-HAA complex with the AAGlyB-**1**-HAA complex, it can be seen that the C terminus in the latter structure fails to form the short α-helix, and neither His-348 nor Arg-352 interacts with the acceptor or the donor analogue (Fig. 8*A*). Instead, these key residues are pointing in the opposite direction, into the solvent, making the electron density of their side chains ill defined, and with elevated isotropic B-factors (Fig. 7, *A* and *B*). In this alternative conformation the main chain of Val-351 now forms two hydrogen bonds to the formylthienyl substituent of **1**, and Asn-353 is forming hydrogen bonds to the backbone of residue Trp-181 as well as the side chain of Gln-182 in the internal loop (Fig. 8*B*). These additional molecular interactions provide, for the first time, a structural explanation for the tight binding of **1** to AAGlyB.

##### AAGlyB Structures in Complex with 2 and 2-HAA

In the AAGlyB-**2** and AAGlyB-**2**-HAA complexes where the 5-substituent of the donor analogue is a phenyl group, the electron density is well defined throughout most of the only chain in the AAGlyB-**2** structure and chain A and B in the AAGlyB-**2**-HAA structure. In the AAGlyB-**2**-HAA structure, upon binding of the acceptor, the internal loop also goes from a disordered state in the “donor-only” complex to an ordered closed conformation with Trp-181 stacking to the phenyl substituent of **2** (Fig. 5, *C* and *D*). However, compared with the AAGlyB-**1**-HAA complex the internal loop of the B chain of AAGlyB-**2**-HAA is slightly less ordered, and residues 196–198 are missing entirely. Furthermore, in the AAGlyB-**2**-HAA structure the C terminus of both chain A and B in the asymmetric unit does not fold over the binding site in the same way as seen in chain A of the AAGlyB-**1**-HAA complex and is only visible until residue 346. Because the AAGlyB-**2**-HAA structure belongs to the same space group and has the same unit cell parameters as the AAGlyB-**1**-HAA structure, this indicates that crystal packing is not the reason for the less ordered C terminus and internal loop. Also, the lack of electron density of the C terminus cannot be explained by poor data resolution because AAGlyB-**2**-HAA diffracted to 1.7 Å whereas AAGlyB-**1**-HAA diffracted to only 1.9 Å. Therefore, the reason for the destabilization of the C terminus and of the internal loop in chain B in the presence of **2** is likely the lack of hydrogen bonding to Val-351, of the type observed for the formylthienyl substituent in **1**. Finally, as for the AAGlyB-**1** structure, the AAGlyB-**2** shows well defined density for the galactose in the tucked under conformation (Fig. 3*C*), whereas in the AAGlyB-**2**-HAA structure the galactose is not visible except for a few blobs extending out from the nucleotide β-phosphate (Fig. 3*D*). This suggests that, although our enzyme kinetics results show a lower turnover number for compound **2** compared with compound **1**, and although the C terminus seems to be incapable of folding over the active site, the enzyme is still slowly hydrolyzing the donor derivative when soaked into the crystal.

## DISCUSSION

We report herein important new structural and enzymological information on the blood group glycosyltransferase AAGlyB, an enzyme with many characteristic features for the glycosyltransferase family in general. These new results allow us to address the open questions raised by the original AAGlyB-**1** structure (8).

Generally, we see that when soaking native donor substrate into these crystals together with HAA acceptor the internal flexible loop and the C terminus fold over the active site and are stabilized by a crucial stacking interaction of Arg-352 and Trp-181 (5, 8). This conformational change corresponds to the transition from the open to the closed conformation during the catalytic cycle. This is a general mechanistic feature of many glycosyltransferases which is required for full catalytic activity (7). The new structures provide the first direct evidence for our hypothesis that the presence of the additional 5-substituent in donor analogues **1** and **2** disrupts this Arg-352/Trp-181 stabilizing interaction and, as a consequence, directly affects the conformational changes that set the scene for the glycosyl transfer reaction.

The structure of the AAGlyB-**1**-HAA complex with a complete flexible loop and C terminus reveals, for the first time, a new, “pseudo-closed” conformation for this enzyme. The new conformation of the C terminus differs from the known closed conformation with donor and acceptor by not forming the same short α-helix and hydrogen bonds as seen in the AAGlyB-UDP-HAA structure. Furthermore, it is different from the open conformation of the C terminus recently shown in a novel GTB-inhibitor complex (27), which also does not form the short α-helix. Interestingly, the unraveling of the α-helix disrupts at least 5–6 main chain hydrogen bonds in the helix, which is presumably highly energetically unfavorable. These findings exemplify rather dramatically the considerable conformational plasticity of GTs.

The new structures with donor and acceptor bound reveal the relative orientation of the internal loop to the donor analogue, and show π-π stacking as another important binding motif for donor analogues. Instead of Trp-181 in the internal loop stacking to Arg-352 in the C terminus, the alternative conformation is characterized by a direct stacking interaction between Trp-181 and the 5-substituent of the donor analogue. This new folding mode is further stabilized by the formation of critical hydrogen bonds between the C terminus and the formyl group of donor analogue **1** as well as the flexible loop.

Although a less effective donor substrate than UDP-Gal, the base-modified donor analogues retain some residual substrate activity. Our results suggest that this pseudo-closed conformation allows the transfer of the sugar, albeit at a slower rate than the native, closed conformation, and could therefore be the reason for the small, residual turnover observed for AAGlyB toward **1**. This is consistent with the observation that for donor analogue **2**, with a much reduced substrate activity (*k*_cat_), neither the alternative folding mode, seen for AAGlyB-**1**-HAA, nor the classical closed conformation, seen for AAGlyB-UDP-HAA, could be observed. The lack of the folded-over flexible loop and the additional hydrogen bonds in the case of **2** may also explain why this UDP-Gal derivative binds less tightly to the donor binding site than **1**, in agreement with the higher *K_m_* and *K_i_* values of **2** compared with **1**.

Although a slightly weaker GT inhibitor than **1**, which is one of the most potent GalT inhibitors to date, it is arguable that this profile makes **2** a particularly suitable candidate for cellular applications, where lack of substrate activity is desirable. The alternative folding mode may therefore represent a unique conformation of AAGlyB, and possibly other GTs, which can be targeted in GT inhibitor design, similar to the DFG conformation in kinases (28). The development of such novel GT inhibitors will be aided further by the resolution, for the first time, of the internal flexible loop and the C terminus in the active site of AAGlyB in the presence of base-modified donor analogues.

## Footnotes

3 The abbreviations used are:

GT: glycosyltransferase
CE: capillary electrophoresis
DA: 3-deoxy-Gal-H antigen acceptor
GalT: galactosyltransferase
GTA: human ABO(H) blood group α-(1→3)-*N-*acetylgalactosaminyltransferase
GTB: human ABO(H) blood group B α-(1→3)-galactosyltransferase
HAA: H antigen acceptor α-l-Fuc-(1→2)-β-d-Gal-*O*-R
PDB: Protein Data Bank.
